# Methionine Metabolism Alters Oxidative Stress Resistance *via* the Pentose Phosphate Pathway

**DOI:** 10.1089/ars.2015.6516

**Published:** 2016-04-01

**Authors:** Kate Campbell, Jakob Vowinckel, Markus A. Keller, Markus Ralser

**Affiliations:** ^1^ Department of Biochemistry and Cambridge Systems Biology Centre, University of Cambridge, Cambridge, United Kingdom.; ^2^ The Francis Crick Institute Mill Hill Laboratory, London, United Kingdom.

## Abstract

Nutrient uptake and metabolism have a significant impact on the way cells respond to stress. The amino acid methionine is, in particular, a key player in the oxidative stress response, and acting as a reactive oxygen species scavenger, methionine is implicated in caloric restriction phenotypes and aging. We here provide evidence that some effects of methionine in stress situations are indirect and caused by altered activity of the nicotinamide adenine dinucleotide phosphate (NADPH) producing oxidative part of the pentose phosphate pathway (PPP). In *Saccharomyces cerevisiae*, both methionine prototrophic (*MET15*) and auxotrophic (*met15*Δ) cells supplemented with methionine showed an increase in PPP metabolite concentrations downstream of the NADPH producing enzyme, 6-phosphogluconate dehydrogenase. Proteomics revealed this enzyme to also increase in expression compared to methionine self-synthesizing cells. Oxidant tolerance was increased in cells preincubated with methionine; however, this effect was abolished when flux through the oxidative PPP was prevented by deletion of its rate limiting enzyme, *ZWF1*. Stress resistance phenotypes that follow methionine supplementation hence involve the oxidative PPP. Effects of methionine on oxidative metabolism, stress signaling, and aging have thus to be seen in the context of an altered activity of this NADP reducing pathway. *Antioxid. Redox Signal.* 24, 543–547.

## Introduction

The ability of cells to counteract oxidative stress is fundamental for survival in an ever changing environment and is implicated in growth and aging (3, 5). Metabolism is of importance for cellular tolerance to oxidants, providing the reducing power for the antioxidant machinery, including the glutathione and thioredoxin systems, and acting as a main source of intracellular oxidants itself (3, 4, 7). A central player in stress resistance is the amino acid methionine. Methionine is a direct target of reactive oxygen species (ROS), as its sulfur can be oxidized to sulfoxide; furthermore, *via* repair of methionine sulfoxide through methionine sulfoxide reductases, methionine can act as scavenger and protect cells from oxidative stress (2). Recent studies analyzing proteomic methionine usage show this amino acid to be enriched in protein at sites of major free radical production, such as the mitochondrial respiratory chain (6). Methionine has therefore been exploited by cells, acting as an ROS scavenger to protect proteins from oxidation, and has been implicated in caloric restriction phenotypes and aging (2, 3).

Genetic experiments have connected methionine biosynthesis to the pentose phosphate pathway (PPP), a pathway with essential roles in the antioxidative metabolism (5, 7). This connection appears to be predominantly caused by the redox cofactor nicotinamide adenine dinucleotide phosphate (NADP). *De novo* methionine biosynthesis, *via* the assimilation of inorganic sulfate, requires three molecules of NADPH per molecule of methionine, and the PPP is key to regenerating NADPH and maintaining redox balance (7). Two PPP enzymes responsible for NADPH production are glucose 6-phosphate dehydrogenase (G6PDH) and 6-phosphogluconate dehydrogenase (6PGDH). When budding yeast is deleted for G6PDH (*ZWF1*), the rate limiting enzyme of this NADPH producing oxidative PPP, cells are no longer able to synthesize methionine and become methionine auxotrophs (8). Growth of *zwf1*Δ cells therefore depends on supplementation with organic sources such as cysteine and methionine (8).

Innovation Methionine plays an essential role in oxidant resistances as it can be oxidized and has been implicated in caloric restriction and aging. We observed altered activity in the oxidative branch of the pentose phosphate pathway (PPP) after increasing the supplementation of methionine, detecting elevated levels of PPP metabolites, and increased abundance of the nicotinamide adenine dinucleotide phosphate (NADPH) producing enzyme 6-phosphogluconate dehydrogenase (6PGDH). Methionine preincubation also increased cellular tolerance to the thiol oxidizing agent diamide in dependency of the oxidative pentose phosphate. At least some oxidative stress resistance phenotypes caused by methionine hence involve the PPP.

## Results and Discussions

**FIG. 1.**
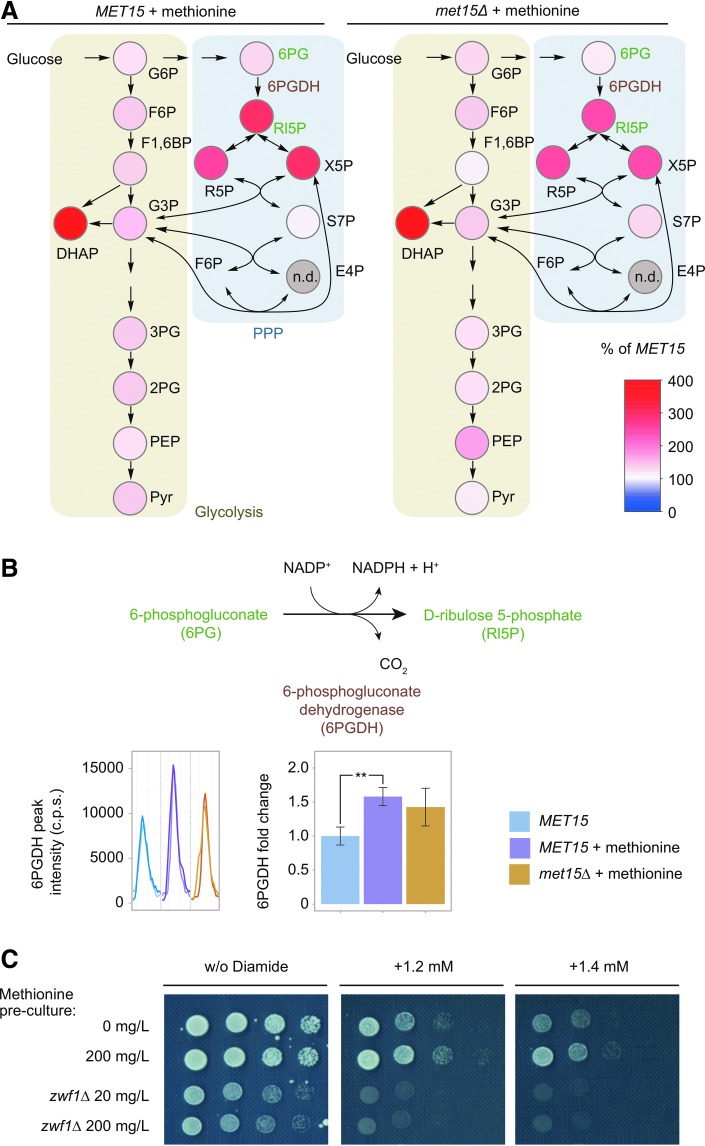
**Methionine supplementation alters intracellular PPP activity**. **(A)** Methionine exposed wild-type (*MET15*) and *met15*Δ cells have increased concentrations of PPP metabolites (*n* = 3). Absolute metabolite concentrations are shown in Table 1. Glycolysis: G6P, glucose 6-phosphate; F6P, fructose 6-phosphate; F1,6BP, fructose 1,6-bisphosphate; DHAP, dihydroxyacetone phosphate; G3P, glyceraldehyde 3-phosphate; 3PG, 3-phosphoglycerate; 2PG, 2-phosphoglycerate; PEP, phosphoenolpyruvate; Pyr, pyruvate. Pentose phosphate pathway: 6PG, 6-phosphogluconate; Rl5P, ribulose 5-phosphate; R5P, ribose 5-phosphate; X5P, xylulose 5-phosphate; S7P, sedoheptulose 7-phosphate; E4P, erythrose 4-phosphate. **(B)** *Top*: Reaction scheme of 6-phosphogluconate (6PG) to ribulose 5-phosphate (Rl5P) catalyzed by 6-phosphogluconate dehydrogenase (6PGDH), yielding NADPH. 6PG and Rl5P are highlighted in green and 6PGDH in brown, here and in **(A)**. *Bottom*: The expression level of 6PGDH in wild-type (*MET15*) as well as in methionine supplemented wild-type and *met15*Δ cells, as determined by SWATH-MS. *Bottom left*: Reconstruction of SWATH-MS chromatographic spectra in Skyline; shown are two transitions of the representative 6PGDH peptide DYFGAHTFR. *Bottom center*: Expression level (fold change to wild type) of 6PGDH. c.p.s., counts per second. *n* = 3, error bars, ± SD. **(C)** Increased resistance of wild-type cells (*ZWF1*) to diamide upon methionine supplementation. This phenotype is lost in cells deleted for the rate-limiting oxidative PPP enzyme G6PDH (z*wf1*Δ). PPP, pentose phosphate pathway. G6PDH, glucose 6-phosphate dehydrogenase. To see this illustration in color, the reader is referred to the web version of this article at www.liebertpub.com/ars

We hence questioned whether the effect of methionine on the PPP was related to the stress response. Direct NADPH quantification in yeast provides limited information about the PPP as (i) a major fraction of NADP(H) is localized to the vacuole or protein bound, (ii) different NADPH sources exist, and (iii) NADPH is readily oxidized upon cell lysis (4). We therefore addressed this problem by combining mass spectrometry-based metabolite quantification, protein quantification, and genetics. First, PPP metabolite abundance was determined using a targeted LC-MS/MS method, which gives absolute quantities for the majority of PPP metabolites (Table 1). Wild-type cells supplemented with methionine showed a distinct increase in PPP metabolites downstream of the NADPH producing enzyme 6PGDH (Fig. 1A, left panel). Auxotrophic yeast, unable to synthesize methionine by themselves, possessed similar concentrations confirming that the effect is mediated by methionine supplementation (Fig. 1A, right panel). Next, protein expression was analyzed by SWATH-MS, a target proteomics method based on LC/MS-MS (9). We detected a clear upregulation of 6PGDH in methionine-supplemented cells (Fig. 1B). Methionine treatment thus increases both metabolite concentration and enzyme expression in the oxidative PPP.

**Table 1.** Absolute Quantification of PPP Metabolites

*Compound name*	*Compound abbreviation*	YSBN5 *(μ*M*)*	YSBN5 + *methionine* (*μ*M)	*(met15Δ)* + *methionine* (*μ*M*)*
Pyruvate	Pyr	190.13 ± 19.31	241.95 ± 17.87	214.23 ± 9.15
Glucose 6-phosphate/fructose 6-phosphate	G6P/F6P	160.50 ± 3.66	188.38 ± 11.54	194.43 ± 4.50
Sedoheptulose 7-phosphate	S7P	126.10 ± 15.24	138.53 ± 8.73	153.80 ± 21.04
Ribose 5-phosphate	R5P	2.73 ± 0.15	5.55 ± 1.03	5.50 ± 1.71
Fructose 6-phosphate	F6P	94.90 ± 6.10	123.90 ± 11.92	123.23 ± 24.97
Glyceraldehyde 3-phosphate	G3P	8.27 ± 0.23	10.95 ± 0.64	10.67 ± 0.60
Xylulose 5-phosphate/ribulose 5-phosphate	X5P/Rl5P	9.20 ± 1.04	21.65 ± 4.31	18.10 ± 2.76
Dihydroxyacetone phosphate	DHAP	4.97 ± 1.66	17.48 ± 5.72	16.33 ± 9.15
6-Phosphogluconate	6PG	43.03 ± 1.71	51.90 ± 2.97	48.93 ± 0.92
2-Phosphoglycerate/3-phosphoglycerate	2-PG/3-PG	69.30 ± 10.37	89.50 ± 8.46	81.23 ± 8.02
Phosphoenolpyruvate	PEP	1.37 ± 0.32	1.63 ± 0.15	2.03 ± 0.38
Fructose 1,6-bisphosphate	F1,6BP	274.80 ± 20.22	343.08 ± 13.15	294.27 ± 19.55

Absolute quantification of PPP metabolites was conducted on the prototrophic yeast strain YSBN5 (*MET15)* ± methionine supplementation and its *MET15* knockout derivative (*met15*Δ) grown with methionine supplementation (*n* = 3). Error = ±SD.

PPP, pentose phosphate pathway.

Finally, we tested for a causal relationship between methionine and the PPP by testing oxidant resistance upon methionine supplementation. The thiol oxidizing agent diamide was considered ideal for testing PPP-dependent oxidant resistance, as cells have increased resistance to this oxidant when PPP activity is increased; however, in contrast to hydrogen peroxide or other peroxides, diamide does not block glycolysis or activate the PPP directly (5). When methionine supplementation was increased from 0 to 200 mg/L, resistance to diamide was increased (Fig. 1C). The upregulated 6PGDH cannot be fully deleted in yeast as it is an essential gene; therefore, to prevent flux in the oxidative PPP, *ZWF1* was deleted instead. In *zwf1*Δ cells, methionine no longer increased diamide tolerance (Fig. 1C).

Stress resistance phenotypes that follow methionine supplementation hence involve the oxidative PPP. Effects of methionine on oxidative metabolism, stress signaling, and aging have thus to be seen in the context of an altered activity of this NADPH providing pathway.

## Notes

### Yeast strains, plasmids, and growth media

All yeast strains and plasmids used are listed in Table 2. For metabolomic and proteomic studies, YSBN5, a prototrophic haploid variant of *Saccharomyces cerevisiae* S288c, was used alongside its *met15*Δ derivative. Deletion of *MET15* in this strain was performed by homologous recombination using a *kanMX* marker (Table 3). To determine the effect of methionine supplementation on oxidative tolerance, BY4741 complemented with the centromeric vector (minichromosome) pHLUM (Addgene ID #40276) was used alongside its *zwf1*Δ derivative. Yeast was cultivated if not otherwise indicated at 30°C, in minimal supplemented synthetic media (SM; YNB yeast nitrogen base [6.8g/L; Sigma]), with 2% glucose (Sigma) as the carbon source.

**Table 2.** Strains and Plasmids Used in This Study

*Name*	*Description*
Strains	
YSBN5	*MATa*, FY3 ho::Ble
YSBN5 *met15*Δ	YSBN5 *met15*::kanMX4
BY4741	MATa, *his3Δ1 leu2Δ0 met15Δ0 ura3Δ0* (ATCC^®^ 201388™)
BY4741 *zwf1*Δ	BY4741 *zwf1*::kanMX4
Plasmids	
pHLUM	Yeast centromeric vector with *HIS3*, *URA3*, *LEU2*, and *MET15* markers (minichromosome). (Addgene number: 40276)

**Table 3.** Oligonucleotides Used to Generate Ysbn5 *Met*15Δ by Homologous Recombination

*Name*	*Sequence*
Met15_fw	GTCAGATACATAGATACAATTCTATTACCCCCATCCATACAAGCTTGCCTCGTCCCCGCCGGGTCA
Met15_rv	GAGAAAGTAGGTTTATACATAATTTTACAACTCATTACGCACACTCGACACTGGATGGCGGCGTTAGTATC

### Quantification of glycolytic and PPP metabolites by LC-MS/MS

Intracellular levels of sugar phosphates were quantified using a previously published LC-MS/MS method (1). 7.5 OD_595_ units of cells (YSBN5 *MET15* ± methionine [20 mg/L] or YSBN5 *met15*Δ + methionine [20 mg/L]) were harvested in the exponential growth phase (OD_595_ = 1.5 ± 0.05) following cold methanol quenching. Cell pellets were resuspended in 200 μL of extraction buffer (75:25 [v/v] acetonitrile:methanol, 0.2% formic acid) and lysed during three FastPrep-24 (MP Biomedicals) cycles for 20 s at 6.5 m/s. After centrifugation for 5 min at 16,000 *g*, the pellet was extracted again with 200 μL ultra high pressure liquid chromatography-grade water. Combined supernatants from both extractions were evaporated in a Concentrator plus SpeedVac (Eppendorf), resuspended in 100 μL 7% acetonitrile, centrifuged, and submitted to LC-MS/MS analysis. One microliter of the metabolite extract was injected on a C8 column (ZORBAX SB-C8 Rapid Resolution HD, 2.1 × 100 mm, 1.8 μm (Agilent); column temperature: 20°C) for liquid chromatography separation (Agilent 1290). Water/acetonitrile mixtures (A: 10% [v/v] acetonitrile, B: 50% [v/v] acetonitrile) that were used as mobile phases contained 750 mg/L octylammoniumacetate as the ion pairing reagent. After 3.5 min of isocratic flow at 12% acetonitrile, a 2.5-min gradient to 38% acetonitrile was used to elute the sugar phosphate analytes, followed by a washing step to 42% acetonitrile for 0.5 min and re-equilibration at starting conditions. Quantification was achieved with an online coupled triple quadrupole mass spectrometer (Agilent 6460) operating in the selective reaction monitoring (SRM) mode. Identification of metabolites was ensured by comparison of retention times and fragmentation patterns with commercially available standards. For each individual compound, optimal analytical conditions (SRM transitions, ionization, and fragmentation energies) were determined separately. MassHunter Workstation software package (Agilent) was used for data analysis. Repeatedly recorded external calibration curves were used to calculate absolute metabolite concentrations. The SRM transitions used are shown in Table 4.

**Table 4.** SRM Transitions for Quantification of Sugar Phosphates by LC-SRM

*Compound name*	*Compound abbreviation*	*Sum formula*	*SRM transition*	*Fragmentor (V)*	*Collision energy (V)*	*Polarity*
Glucose	Glu	C_6_H_12_O_6_	179.0 > 89.0	70	1	−
Pyruvate	Pyr	C_3_H_4_O_3_	87.0 > 43.0	55	3	−
Sedoheptulose 7-phosphate	S7P	C_7_H_15_O_10_P	289.0 > 97.0	100	12	−
Glucose 6-phosphate	G6P	C_6_H_13_O_9_P	259.0 > 97.0	100	12	−
Xylulose 5-phosphate/ribulose 5-phosphate	X5P/Rl5P	C_5_H_9_O_8_P	229.0 > 97.0	85	12	−
Fructose 6-phosphate	F6P	C_6_H_13_O_9_P	259.0 > 97.0	100	12	−
Erythrose 4-phosphate	E4P	C_4_H_9_O_7_P	199.0 > 97.0	70	6	−
Glyceraldehyde 3-phosphate	G3P	C_3_H_7_O_6_P	169.0 > 97.0	70	5	−
Ribose 5-phosphate	R5P	C_5_H_9_O_8_P	229.0 > 97.0	85	12	−
Dihydroxyacetone phosphate	DHAP	C_3_H_7_O_6_P	169.0 > 97.0	70	5	−
6-Phosphogluconate	6PG	C_6_H_13_O_10_P	275.0 > 97.0	100	18	−
2-Phosphoglycerate/3-phosphoglycerate	2-PG/3-PG	C_3_H_7_O_7_P	185.0 > 97.0	75	11	−
Phosphoenolpyruvate	PEP	C_3_H_5_O_6_P	167.0 > 79.0	50	7	−
Fructose 1,6-bisphosphate	F1,6BP	C_6_H_14_O_12_P_2_	339.0 > 97.0	175	16	−

SRM, selective reaction monitoring.

### Proteomic analysis of PPP enzyme abundance by SWATH-MS

Analysis of PPP enzyme abundances was performed using SWATH-MS as previously described. In short, cells equaling 10 OD_595_ of an exponentially growing yeast culture (YSBN5 *MET15* ± methionine [20 mg/L] or YSBN5 *met15*Δ + methionine [20 mg/L]) were harvested and tryptic peptides prepared in accordance with the RapidACN method (9). LC-MS/MS analysis was performed on a Tandem Quadrupole Time-of-Flight mass spectrometer (AB/Sciex TripleTOF5600) equipped with a Turbo V ion source and 25 μm emitter needles (AB/Sciex), on line coupled to a HPLC system (Eksigent nanoLC 425) operating in the microflow mode. Chromatographic separation was achieved by direct injection of 10 μg yeast digest supplemented with 0.5× HRM-Kit (Biognosys) onto an analytical column (Eksigent ChromXP, 3 μm, C18CL, 300Å, 150 × 0.3 mm) followed by a linear gradient (0–28% acetonitrile/0.1% formic acid in 26 min) at a flow rate of 3 μL/min. Data acquisition was performed with 29 × 16 m/z SWATH windows, a range of 400–850 m/z, and 40 ms accumulation time resulting in 1.2 s cycle time. Extracted ion chromatograms were then extracted with the help of Spectronaut software (version 5.0.5900; Biognosys) by querying the data with an ion library, constructed by operating the mass spectrometer in the data-dependent acquisition mode and repeatedly injecting tryptic yeast digests prefractionated using high pH reverse phase chromatography. The chromatographic setup for the ion library was as outlined above. Data were validated by manually removing interfering fragment ions using Skyline software, and the three most abundant fragment ions were used for calculating peptide fold changes. To correct for amount of sample injected, data were normalized using the fold change of glyceraldehyde-3-phosphate dehydrogenase (EC 1.2.1.12; *TDH1*) as a control. Protein fold changes were calculated by averaging three peptide fold changes per protein. Transitions of peptide DYFGAHTFR for 6PGDH (EC 1.1.1.44; *GND1*) taken from SWATH chromatographic spectra in Skyline were then illustrated with R package “ggplot2.” Fold change in protein abundance for YSBN5 *MET15* and *met15*Δ strains + methionine was then based on protein abundance for YSBN5 *MET15* strains untreated.

### Oxidative stress for prototroph and *zwf1Δ* strains

For the oxidant tolerance test following methionine treatment, BY4741 *ZWF1* and *zwf1*Δ strains, complemented with the centromeric vector pHLUM, were grown for two nights in SM ± methionine (*ZWF1* with 0 and 200 mg/L, and *zwf1Δ* with 20 and 200 mg/L of L-methionine [Sigma], respectively, as the *zwf1*Δ strain is auxotrophic for organic sulfur (8)). Cell cultures were then normalized to 3.6e+06 cells in 200 μL SM and spotted in 1:5 serial dilutions on SM solid media with l-methionine (200 mg/L) ± diamide (Sigma). Growth was documented after 3 days incubation at 30°C.

## Abbreviations Used

6PGDH: 6-phosphogluconate dehydrogenase
G6PDH: glucose 6-phosphate dehydrogenase
NADPH: nicotinamide adenine dinucleotide phosphate
PPP: pentose phosphate pathway
ROS: reactive oxygen species
SM: synthetic media
SRM: selective reaction monitoring

## References

[1] Keller MA, Turchyn AV, and Ralser M Non-enzymatic glycolysis and pentose phosphate pathway-like reactions in a plausible Archean ocean. Mol Syst Biol 10: 725, 2014 24771084 10.1002/msb.20145228 PMC4023395

[2] Luo S and Levine RL Methionine in proteins defends against oxidative stress. FASEB J 23: 464–472, 2009 18845767 10.1096/fj.08-118414 PMC2630790

[3] Pamplona R and Barja G Mitochondrial oxidative stress, aging and caloric restriction: the protein and methionine connection. Biochim Biophys Acta 1757: 496–508, 2006 16574059 10.1016/j.bbabio.2006.01.009

[4] Pollak N, Dölle C, and Ziegler M The power to reduce: pyridine nucleotides—small molecules with a multitude of functions. Biochem J 402: 205–218, 2007 17295611 10.1042/BJ20061638 PMC1798440

[5] Ralser M, Wamelink MM, Kowald A, Gerisch B, Heeren G, Struys EA, Klipp E, Jakobs C, Breitenbach M, Lehrach H, and Krobitsch S Dynamic rerouting of the carbohydrate flux is key to counteracting oxidative stress. J Biol 6: 10, 2007 18154684 10.1186/jbiol61 PMC2373902

[6] Schindeldecker M and Moosmann B Protein-borne methionine residues as structural antioxidants in mitochondria. Amino Acids 47: 1421–1432, 2015 25859649 10.1007/s00726-015-1955-8

[7] Stincone A, Prigione A, Cramer T, Wamelink MMC, Campbell K, Cheung E, Olin-Sandoval V, Gruening N, Krueger A, Tauqeer Alam M, Keller MA, Breitenbach M, Brindle KM, Rabinowitz JD, and Ralser M The return of metabolism: biochemistry and physiology of the pentose phosphate pathway. Biol Rev Camb Philos Soc 90: 927–963, 2014 10.1111/brv.12140 PMC4470864 25243985

[8] Thomas D, Cherest H, and Surdin-Kerjan Y Identification of the structural gene for glucose-6-phosphate dehydrogenase in yeast. Inactivation leads to a nutritional requirement for organic sulfur. EMBO J 10: 547–553, 1991 2001672 10.1002/j.1460-2075.1991.tb07981.x PMC452682

[9] Vowinckel J, Capuano F, Campbell K, Deery MJ, Lilley KS, and Ralser M The beauty of being (label)-free: sample preparation methods for SWATH-MS and next-generation targeted proteomics. F1000Res 2: 272, 2013 24741437 10.12688/f1000research.2-272.v2 PMC3983906

